# Highly-Ordered PdIn Intermetallic Nanostructures Obtained from Heterobimetallic Acetate Complex: Formation and Catalytic Properties in Diphenylacetylene Hydrogenation

**DOI:** 10.3390/nano8100769

**Published:** 2018-09-28

**Authors:** Igor S. Mashkovsky, Pavel V. Markov, Galina O. Bragina, Galina N. Baeva, Alexander V. Rassolov, Ilya A. Yakushev, Michael N. Vargaftik, Alexander Yu. Stakheev

**Affiliations:** 1 N. D. Zelinsky Institute of Organic Chemistry, Russian Academy of Sciences, Leninsky Prospect 47, 119991 Moscow, Russia; im@ioc.ac.ru (I.S.M.); markovp@mail.ru (P.V.M.); bragina@ioc.ac.ru (G.O.B.); bg305@ioc.ac.ru (G.N.B.); brandon_hitt@mail.ru (A.V.R.); 2 N. S. Kurnakov Institute of General and Inorganic Chemistry, Russian Academy of Sciences, Leninsky Prospect 31, 119991 Moscow, Russia; cs68@mail.ru (I.A.Y.); wahr36@gmail.com (M.N.V.)

**Keywords:** Pd_1_In_1_, intermetallic nanoparticles, selective hydrogenation, diphenylacetylene, heterobimetallic complex

## Abstract

Formation of PdIn intermetallic nanoparticles supported on α-Al_2_O_3_ was investigated by X-ray powder diffraction (XRD), transmission electron microscopy (TEM), and hydrogen temperature-programmed desorption (H_2_-TPD) methods. The metals were loaded as heterobimetallic Pd(μ-O_2_CMe)_4_In(O_2_CMe) complex to ensure intimate contact between Pd and In. Reduction in H_2_ at 200 °C resulted in Pd-rich PdIn alloy as evidenced by XRD and the disappearance of Pd hydride. A minor amount of Pd_1_In_1_ intermetallic phase appeared after reduction at 200 °C and its formation was accomplished at 400 °C. Neither monometallic Pd or in nor other intermetallic structures were found after reduction at 400–600 °C. Catalytic performance of Pd_1_In_1_/α-Al_2_O_3_ was studied in the selective liquid-phase diphenylacetylene (DPA) hydrogenation. It was found that the reaction rate of undesired alkene hydrogenation is strongly reduced on Pd_1_In_1_ nanoparticles enabling effective kinetic control of the hydrogenation, and the catalyst demonstrated excellent selectivity to alkene.

## 1. Introduction

Nowadays, bimetallic metal-supported catalysts are widely used for a number of industrial and laboratory relevant reactions. These catalysts usually comprise a noble metal (Pd, Pt) and a second metal, which is added as modifier to improve catalytic performance [1]. In bimetallic catalysts two metals can be present as alloys, ‘core–shell’ structures or metal–oxide composites [2]. Substitution solid-solution alloys consist of metals with similar atomic size, electronic character, and a crystal structure identical to that of the parent metals with random atomic arrangements. However, many alloys tend to agglomerate, segregate, or phase separate under reaction conditions, which deteriorates their catalytic characteristics [3]. Moreover, unlike homogeneous catalysts, the presence of a variety of active sites on their surface significantly reduces selectivity in catalytic reactions. Therefore, in recent years, highly-ordered intermetallic compounds (IMCs) have attracted significant attention as a possible alternative to substitutional alloys in catalysis [2,4,5,6,7,8]. By general definition intermetallic compound is a chemical compound consisting of two or more metallic elements, which exhibits a highly ordered crystal structure different from the constituting elements [1]. In contrast to substituted alloys IMCs possess highly-ordered atomic-level structure in the bulk, and on the surface. The specific combination of covalent and ionic interactions and the presence of conducting electrons results in peculiar combination and homogeneity of electronic and crystal structures, providing unique adsorption and catalytic properties of IMCs [1,2,9]. Another advantage of IMCs is its thermodynamic stability in comparison with traditional solid-solution alloys [2]. Furthermore, as it was proposed in [2], highly ordered intermetallic structures are suitable to design theoretical models for computational studies. Thus, intermetallics are promising materials for developing well-structured catalysts introducing novel concepts of fundamental catalysis. By selecting intermetallic compounds with suitable structures, the properties of active sites can be fitted to the reaction needs.

It is important that application of IMCs makes it possible to obtain highly ordered structures on the surface of nanoparticles. Thus, a fundamental study of Pd-Ga IMCs revealed its enhanced stability and extremely high selectivity in gas-phase acetylene hydrogenation. For interpretation of the unique selectivity of PdGa systems authors proposed a concept of site-isolated catalysts, which is closely related to the concept of a single-atom catalyst [10]. Isolation of Pd atoms by neighboring Ga atoms due to formation of IMCs was confirmed by both theoretical calculations and several characterization methods. According to original concept for characterization of PdGa nanoparticles, Kovnir et al. applied quantum-chemical calculations coupled with in situ XPS and FTIR-CO techniques [10]. It was shown that electronic structures of PdGa and Pd metal significantly differs each other and the surface of PdGa nanoparticles contains only isolated Pd atoms surrounded by Ga, which leads to uniformity of active sites similar to that in homogeneous catalysts. Such structure of active sites provides high selectivity and long-term stability in selective acetylene hydrogenation. The latter is probably due to the absence of carbon deposits. Thus, it is well known that the formation of carbonaceous deposits is one of the main reasons for deactivation of monometallic Pd catalysts [10,11,12]. The structure of PdGa IMCs does not have neighbored Pd atoms, which are necessary to form carbon or hydrocarbon deposits. Thus, authors attributed significantly enhanced catalytic stability of PdGa to both geometric and electronic effects [6,10].

Previously, it was shown that the concept of site-isolated catalysts is also applicable for the catalysts containing PdIn IMCs. Our recent data of Diffuse reflectance infrared fourier transform spectroscopy of adsorbed CO (CO-DRIFTS) revealed the presence of isolated palladium atoms surrounded by indium on the surface of PdIn intermetallic nanoparticles [13]. It was also found that PdIn catalysts demonstrate high selectivity in the liquid-phase hydrogenation of internal alkynes comparable with that of the commercial Lindlar catalyst. Remarkably, excellent activity/selectivity characteristics were attained at a significantly lower Pd content [14]. These results are in the good agreement with the data reported by Feng et al. [15]. Using computational chemistry modeling with density functional theory (DFT) authors demonstrated that the PdIn (110) surface is composed of single Pd atom sites isolated by In atoms. Moreover, high selectivity of Pd-In system in gas-phase acetylene semihydrogenation was predicted and confirmed experimentally. The experimental results clearly show that PdIn IMCs with isolated Pd single-atom sites exhibit excellent selectivity to ethylene formation (92% at 90 °C), which is much higher than for monometallic Pd. It was proved that the interaction between Pd and In resulting in PdIn IMCs formation can effectively stabilize the Pd single site and enhance long-term catalyst stability [15]. Our recent publication also revealed excellent selectivity of PdIn IMCs in gas-phase propyne hydrogenation [16].

However, synthesis of a catalyst containing supported IMCs nanoparticles with identical composition and structure is not a trivial task. Conventionally, PdIn supported intermetallic compounds can be obtained via reactive metal-support interaction technique (RMSI), when the oxide support itself is modified with a second metal [1,5,17]. The RMSI term refers to a chemical interaction on the metal–oxide surface under high reduction temperature, which leads to the alloy or IMCs formation. The widely used oxides for catalyst preparation comprise Ga_2_O_3_ [6,10,18], GeO_2_ [7], CeO_2_ [19], ZnO [9,20,21], and In_2_O_3_ [17,22,23]. The main problem using reactive metal-support interaction for the synthesis of supported IMC nanoparticles is that usually a mixture of IMCs with the supported metal (modifier) is formed. Thus for the Pd/ZnO system a mixture of ZnPd, ZnPd_2_, and Zn_3_Pd_2_ was detected after reduction treatment [9,24]. Analogously, the synthesis of supported PdIn IMC via RMSI is complicated by the complex Pd-In phase diagram, exhibiting a number of thermodynamically stable phases [25,26]. Therefore, interaction between In and Pd usually leads to several different IMCs (Pd_3_In_7_, Pd_2_In_3_, PdIn, Pd_5_In_3_, Pd_2_In, and Pd_3_In) [20,23]. Additionally, RMSI method requires specific carrier materials and is not suitable for traditional catalytic supports (SiO_2_, Al_2_O_3_) or metal free supports (C, BN). With these systems, formation of intermetallic compounds can be expected only at reduction temperatures >600 °C [1,5,27]. Therefore, an alternative preparation technique needs to be developed that allows obtaining nanoparticles of IMCs on the surface of different supports.

Wu et al. synthesized silica-supported PdIn intermetallic nanoparticles with different Pd:In ratio by the traditional incipient wetness impregnation [23]. Formation of IMCs was detected by in situ X-ray absorption spectroscopy (XAS) and in situ XRD methods. However, this approach also leads to a mixture of different IMCs structures as indicated by several characterization methods. Fourier-transform infrared spectroscopy of adsorbed CO (FTIR-CO) data shows a complete suppression of the bridged CO adsorption signal for the sample with a large excess of indium (Pd:In = 1:2). Absence of this signal indicates that the nanoparticles are well-ordered PdIn intermetallics with only isolated single-atom Pd sites on the surface [23]. However, large In excess is unfavorable, since it leads to the formation of an oxide shell around the intermetallic nanoparticles core which suppress catalyst activity [7,23].

A convenient way to synthesize supported PdIn IMCs with a pre-defined stoichiometry is to use heterometallic PdIn acetate complex as an IMC precursor [28]. Originally, two metal components are linked together within initial complex structure by strong acetate bridges, thus ensuring intimate bonding of both metals during metal loading and providing uniform stoichiometry of IMC nanoparticles in a final catalyst. This approach was successfully applied earlier [29,30,31,32] for preparation of PdCu and PdAg supported catalysts. PdIn samples were also investigated in our group, however, the formation of PdIn IMC has not been studied in detail and the optimal conditions for the PdIn IMCs formation remain unclear. Therefore, the aim of this work was to investigate the mechanism of IMCs formation with the ultimate goal of obtaining uniform PdIn nanoparticles with a 1:1 stoichiometry without other IMCs phases and investigating the applicability of these systems in the liquid-phase hydrogenation of substituted alkynes. This reaction is of significant importance because its products (cis-/trans-olefins) play a key role as source materials for the food and pharmaceutical industry, domestic chemicals, light-emitting diodes, liquid-crystal displays, etc. [33,34,35,36].

For the catalyst preparation α-Al_2_O_3_ was used as the carrier. The choice was motivated by two main reasons. First, highly-crystalline structure of α-Al_2_O_3_ makes it possible to easily trace a formation of PdIn intermetallic nanoparticles by a conventional XRD method, since α-Al_2_O_3_ diffraction pattern exhibits narrow reflexes that practically do not overlap peaks characteristic of metallic Pd, In, and PdIn. Second, high chemical inertness and a wide-porous structure make this material ideal for utilization in liquid-phase reactions.

## 2. Materials and Methods

### 2.1. Materials

Diphenylacetylene (Sigma-Aldrich, St. Louis, MO, USA, >98%) and *n*-hexane (Merck KGaA, Darmstadt, Germany, 98%) were used without further purification. Gases and gaseous mixtures used for catalyst reduction and catalytic tests were supplied by Linde Gas Rus (Balashikha, Russia): 5%H_2_/Ar, H_2_ (99.9999% grade) and He (99.999% grade). For the catalyst preparation α-Al_2_O_3_ (Alfa Aesar, Haverhill, MA, USA, S_sp_ = 8 m^2^/g) was used as a carrier.

### 2.2. Catalyst Preparation

The 3%Pd-3.2%In/α-Al_2_O_3_ samples were prepared by incipient wetness impregnation of precalcined α-Al_2_O_3_ (air flow, 550 °C, 4 h) with aqueous solution of Pd(μ-O_2_CMe)_4_In(O_2_CMe) complex, followed by drying overnight at room temperature and calcination (air flow, 550 °C, 4 h). To study the PdIn IMCs formation, the samples were reduced at 200, 300, 400, 500, and 600 °C (5% H_2_/Ar, 2 h) and signed as PdIn-200, PdIn-300, PdIn-400, PdIn-500, and PdIn-600 (see Table 1).

### 2.3. Catalyst Characterization

X-ray diffraction (XRD). Powder XRD patterns were obtained on a D8 Advance diffractometer (Bruker, Karlsruhe, Germany; CuKα, Ni filter, LYNXEYE detector, reflection geometry). The peaks identification was performed using the PDF-2-2014 database. The experimental details were reported elsewhere [37].

Transmission electron microscopy (TEM). The microstructure of the samples was studied using an HT7700 instrument (Hitachi, Japan). The images were acquired in the transmitted electron detection mode (bright field imaging) at an accelerating voltage of 100 kV. Before the measurements, powdered samples were supported from an isopropanol suspension onto copper gauzes (O.D. 3 mm) covered with a carbon. The average size of metal particles was determined from the measurement of 150–180 nanoparticles in different areas of TEM images.

Temperature-programmed Pd hydride decomposition (TPHD). Temperature-programmed hydride decomposition (TPHD) was performed in a homemade semiautomatic continuous flow setup equipped with a thermal conductivity detector (TCD), water vapor trap, and a data acquisition and processing unit. The pre-reduced sample (25 mg) was placed in the reactor and dried in Ar flow at 300 °C for 1 h. After that the sample was treated in a 5% H_2_/Ar flow for 15 min at the temperature corresponding to the reduction temperature on the preparation stage. The sample was cooled down to 0 °C and purged with Ar. TPHD analysis was performed at temperature range from 0 to 300 °C with 10 °C/min ramp and the hydrogen evolution was continuously measured by TCD.

### 2.4. Catalytic Tests

The liquid-phase DPA hydrogenation was performed in an homemade autoclave setup equipped with a magnetic stirrer, a gas dosing/probing system, and an electronic pressure sensor for the estimation of the H_2_ absorption. The autoclave was purged with helium to remove the residual air and then the reaction gas H_2_ was introduced into the reactor. The reaction was carried out at ambient temperature and at a stirring speed of 1000 rpm. The intensity of mixing was selected to ensure that the process proceeds in the kinetic region [38]. The hydrogen pressure was set at 10 bar and maintained throughout the experiment. The liquid products were periodically sampled and then analyzed by gas chromatography on a Crystal 5000 instrument (Chromatec, Yoshkar-Ola, Russia) equipped with an HP5-MS column (5% phenyl dimethylsiloxane, 30 m, the internal diameter of 0.25 mm) and a flame ionization detector (FID).

**Figure 1:**
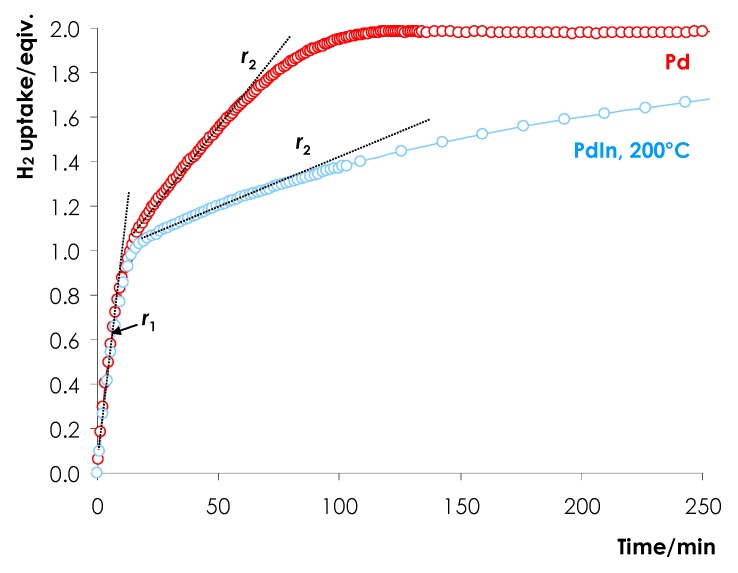
Typical kinetic profiles of H_2_ uptake on the reaction time in the course of the liquid-phase hydrogenation of DPA on the Pd/Al_2_O_3_ and PdIn/Al_2_O_3_ reduced at 200 °C.

The reaction rates *r* (mmol H_2_ g_Cat_^−1^ min^−1^) were determined by evaluating the rate of H_2_ consumption from the dependence of hydrogen uptake on the reaction time (Figure 1) [39].

Hydrogenation of DPA proceeds in two stages: on the first stage alkyne-to-alkene hydrogenation prevails, while on the second stage the main reaction is the alkene-to-alkane hydrogenation. The reaction rate was determined for each stage. The rate of alkyne-to-alkene hydrogenation (*r*_1_) for all samples was evaluated within the range of H_2_ uptake of 0.1–0.4 equiv. H_2_ and compared with the hydrogenation rate determined on the basis of gas chromatography analysis of the reaction mixture after uptake of 0.1 and 0.4 equiv. H_2_. Both results were found to be in a good agreement (deviation is less than +/−2.5%)

The rate of alkene to alkane conversion (*r*_2_) was determined within the uptake range of 1.1–1.3 equiv. H_2_. These results were also verified on the basis of gas chromatography (GC) analysis of the reaction mixture after uptake of the relevant amounts of hydrogen.

The efficiency of the kinetic control of semihydrogenation was evaluated by a ratio between the hydrogenation rates at the first and second stages (*r*_1_/*r*_2_).

Selectivity with respect to stylbene (*S*_St_) as a target product was calculated from GC data using the equation

*S*_St_ = *C*_St_/(*C*_St_ + *C*_DPE_),

where *C*_St_ and *C*_DPE_ are the concentrations of stylbene and diphenylethane (DPE), respectively.

## 3. Results and Discussion

### 3.1. Catalyst Characterization

#### 3.1.1. X-ray Diffraction

**Figure 2:**
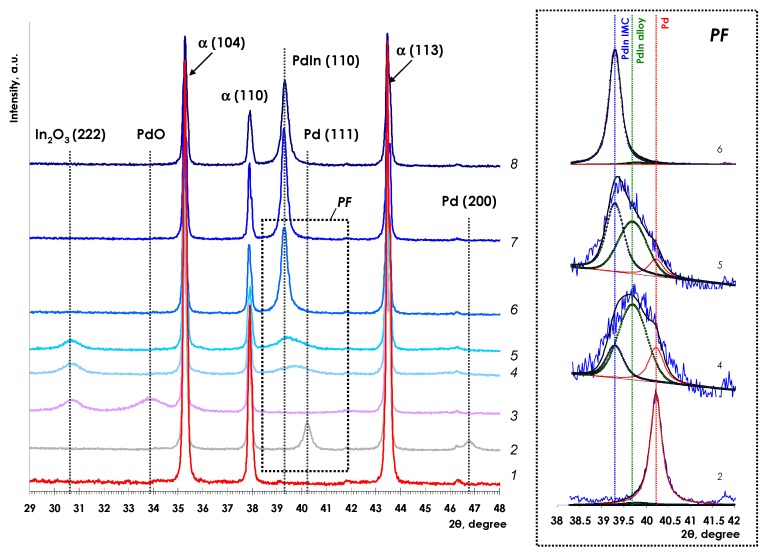
Comparison of XRD patterns for pure α-Al_2_O_3_ (1), monometallic Pd/α-Al_2_O_3_ (2), and PdIn/α-Al_2_O_3_ catalysts unreduced (3) and reduced t 200 °C (4), 300 °C (5), 400 °C (6), 500 °C (7), and 600 °C (8). Insert on the right demonstrates peak fitting (PF) performed for the profiles 2, 4, 5, and 6.

In order to determine the optimal conditions for the formation of PdIn intermetallic nanoparticles Pd–In/α-Al_2_O_3_ sample was subjected to the reductive treatment in 5%H_2_/Ar at 200, 300, 400, 500, to 600 °C. The structural transformations of the PdIn/α-Al_2_O_3_ catalyst depending on the reduction temperature are plotted in Figure 2. XRD patterns of α-Al_2_O_3_, Pd/α-Al_2_O_3_ and initial unreduced PdIn/α-Al_2_O_3_ are also displayed for a comparison.

The parent α-Al_2_O_3_ (Figure 2, pattern 1) exhibits characteristic narrow diffraction peaks at 2θ angles of ca. 35.3, 37.9, and 43.4° indicating a highly-crystalline structure of the carrier [40,41,42]. Additional reflexes typical for α-Al_2_O_3_ at 25.6° (012), 52.6° (024), 57.6° (116), 59.8° (211), 61.3° (018), 66.5° (214), 68.2° and 77° (119) are not shown.

Two additional diffraction peaks were detected for Pd/Al_2_O_3_ (Figure 1, pattern 2) at 2θ of ca. 40.2° and ca. 46.7° attributed to the Pd (111) and Pd (200), respectively [17,43,44,45].

For the unreduced PdIn/α-Al_2_O_3_ catalyst (Figure 2, pattern 3), the XRD patterns reveals two types of oxide phases: In_2_O_3_ (222) at 2θ ca. 30.7° and PdO at 2θ ca. 33.8° [46,47,48]. It should be noted that neither Pd^0^ nor In^0^ typical signals are observed.

After mild reduction of PdIn/α-Al_2_O_3_ catalyst at 200 °C PdO reflexes disappear and a wide reflex appears with maximum at 2θ ca. 39.7° (Figure 2, pattern 4). It is remarkable that the peak maximum is shifted toward lower 2θ in comparison with Pd (111) of Pd/Al_2_O_3_. Peak fitting analysis of its XRD profile (Insert of Figure 2, pattern 4) reveals three reflexes at 2θ~40.2°, 39.7°, and 39.2° (peak area ratio = 0.19:0.62:0.19). The peak at 40.2° is characteristic of Pd(111) indicating the presence of metallic Pd. Appearance of the intensive peak at 2θ~39.7° suggests the formation of Pd-rich alloy in accordance with PdIn phase diagram [26]. Easy formation of Pd-rich alloy even at relatively low temperature agrees with the data reported for Pd/In_2_O_3_ and attributable to the hydrogen activation on Pd^0^, which facilitates In reduction [17,20]. The low-intensive peak at 2θ~39.2° (characteristic of Pd_1_In_1_(110)) suggests the formation of a minor amount of Pd_1_In_1_ IMC. However, the reduction temperature of 200 °C appears to be insufficient for a complete transformation of Pd and In components into well-ordered IMC structure. Moreover, a part of In still remains unreduced as In_2_O_3_ (reflex at 2θ ca. 30.7°).

After reduction at 300 °C intensity of the reflexes at 2θ~40.2° (metallic Pd) and 39.7° (Pd-rich PdIn alloy) decreases, and the peak at 39.2° rises (peak area ratio = 0.44:0.46:0.10) (see insert of Figure 2, pattern 5) indicating intensive transformation of Pd and In components into Pd_1_In_1_ IMC.

After reduction at 400 °C, only an intensive signal at 39.3° remains in the XRD pattern characteristic of Pd_1_In_1_ (110) with CsCl type structure [17]. The intensity and symmetry of the reflex indicates the ordering of PdIn IMCs structure. In addition, the peak at 2θ ca. 30.7° characteristic of indium oxide completely disappears.

These data are in general agreement with the results reported by Lorenz and Neumann [17,20], which revealed formation of Pd_1_In_1_ via RMSI mechanism upon reduction Pd/In_2_O_3_ at 300 °C. However, the formation of well-ordered Pd_1_In_1_ intermetallic nanoparticles over α-Al_2_O_3_ surface requires higher temperature and is accomplished only at 400 °C.

No significant changes in diffraction patterns were observed after following reduction at 500 and 600 °C which indicates that the formation of Pd_1_In_1_ structure was completed at 400 °C. It should be mentioned that, in contrast to Pd-In/α-Al_2_O_3_, intensive formation of In-rich IMC was previously observed upon reduction of Pd/In_2_O_3_ [17,20]. Thus, Pd_2_In_3_ was detected after reduction at 400 °C, followed by the formation of PdIn_3_ [17] and Pd_3_In_7_ [20] at 500 °C. Evidently, in our study, the formation of In-rich IMC is avoided, because the amount of In is limited by the stoichiometry of parent PdIn complex.

#### 3.1.2. Transmitted Electron Microscopy

**Figure 3:**
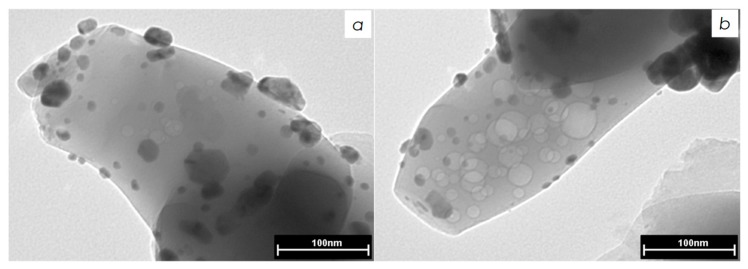
TEM images of different regions (**a**,**b**) of Pd/α-Al_2_O_3_ catalyst, reduced at 500 °C.

Morphology and distribution of Pd and Pd–In nanoparticles were studied by TEM technique. Figure 3 shows the micrographs of the reference Pd/α-Al_2_O_3_ catalysts reduced at 500 °C. The data indicate that the catalyst contains almost spherical well-distributed Pd particles with an average size of ca. 15–20 nm.

**Figure 4:**
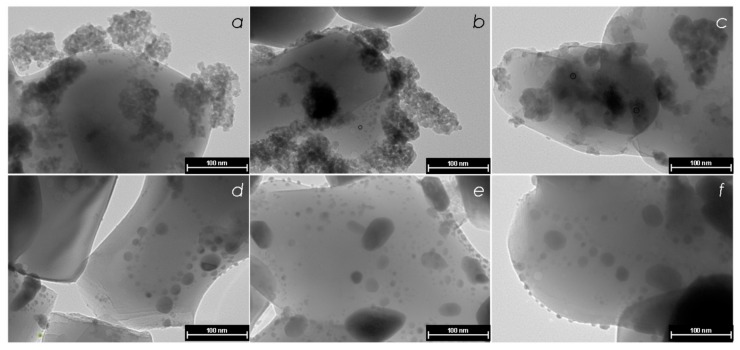
TEM images of the calcined Pd-In/α-Al_2_O_3_ (**a**) and the catalyst reduced at 200 °C (**b**), 300 °C (**c**), 400 °C (**d**), 500 °C (**e**), and 600 °C (**f**).

The TEM image shown in Figure 4a represents the initial unreduced Pd–In/α-Al_2_O_3_ catalyst. Amorphous particles of PdO and In_2_O_3_ oxides can be clearly seen due to strong contrast variation.

After reduction of Pd–In/α-Al_2_O_3_ at 200 °C one can distinguish formation of the nearly spherical contrast particles with average size of ca. 6 nm, surrounded by less contrast amorphous phase (Figure 4b). Taking into account XRD data the contrast species are mostly attributable to Pd-rich alloy nanoparticles (XRD peak at 2θ ca. 39.7°) surrounded by In oxide evidenced by the wide low-intensity reflex 2θ ca. 30.7°. Comparison of the TEM and XRD data for PdIn-200 sample indicates that the reduction of PdO and the formation of Pd^0^ metallic species are accompanied by a reduction of In_2_O_3_, though a significant part of indium oxide remains unreduced.

The following increase in the reduction temperature to 300 °C results in almost complete disappearance of the amorphous In_2_O_3_ species, and the formation of more contrast agglomerates (Figure 4c). The species formed at 300 °C exhibit complex irregular shape indicating the defectiveness of their structure. These data also agree with the XRD data and allow us to conclude that the agglomerates are the species of PdIn alloy with different composition including Pd_1_In_1_ intermetallic nanoparticles and a minor amount of monometallic Pd.

The reduction at 400 °C leads to the complete disappearance of the residual amorphous In_2_O_3_ agglomerates and the formation of metal crystallites with the regular nearly spherical shape (Figure 4d). It is noteworthy that the XRD pattern of this sample reveals only the intensive symmetrical reflex at 2θ = 39.2° characteristic of Pd_1_In_1_ IMC, while the peaks characteristic of metallic Pd and PdIn alloy rich in Pd disappear completely. Thus, the TEM and XRD data point to the formation of Pd_1_In_1_ intermetallic nanoparticles of homogeneous composition with average particle size of ca. 10 nm.

The following increase of reduction temperatures up to 500 and 600 °C significantly change neither micrograph patterns nor the XRD profiles (see Section 3.1.1). Regular spherical particles were observed of ca. 9–12 nm (Figure 4e,f). Additionally, several agglomerates with sizes from 30 to 70 nm can be distinguished in the micrographs of Pd-500 and Pd-600, indicating a sintering of PdIn intermetallic nanoparticles [49].

#### 3.1.3. Temperature-Programmed Pd Hydride Decomposition

It is well-known that Pd is able to absorb a considerable amount of hydrogen due to formation of α and β hydride phases (PdH*_x_*) [50]. Hydrogen from PdH*_x_* structure migrates to the palladium surface in the course of hydrogenation and provokes the complete hydrogenation of the adsorbed substrate thus diminishing the selectivity toward desirable alkene. Formation of PdH*_x_* hydride phases can be inhibited by modification of Pd catalyst with a second metal. Several research groups associate the higher selectivity of bimetallic catalysts in hydrogenation with the suppression of complete hydrogenation involving hydrogen from PdH*_x_* [4,6,50,51].

Due to importance of this factor, a possible formation of Pd hydride in the PdIn/α-Al_2_O_3_ catalysts reduced at different temperatures, and in the reference Pd/α-Al_2_O_3_ was studied by a saturation of the samples with H_2_ followed by temperature-programmed hydride decomposition.

**Figure 5:**
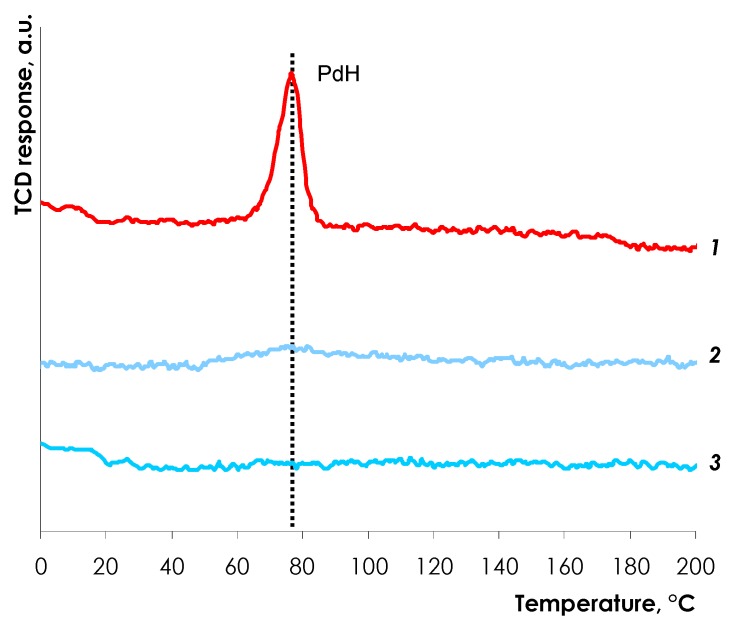
Temperature-programmed hydride decomposition of Pd/α-Al_2_O_3_ (1) and Pd-In/α-Al_2_O_3_ catalysts reduced at 200 °C (2) and 300 °C (3).

As shown in Figure 5, the peak of hydride decomposition is evidently observed for the Pd/Al_2_O_3_ catalyst at the temperature range of 60–85 °C with maximum at ca. 77 °C (profile 1). The data are in good agreement with previously reported results [51,52,53,54]. The ratio of H/Pd was calculated to be 0.32, and this small value can be explained by the relatively low hydrogen partial pressure since 5% H_2_/Ar mixture was used. Moreover, the hydrogen solubility in supported palladium depends on the metal particle size and decreases for smaller Pd particles [51].

In contrast to Pd/Al_2_O_3_, no significant signals in TPHD profiles are detected for the Pd-In/Al_2_O_3_ catalyst. Only traces of H_2_ evolution at 55–85 °C can be discerned for the catalyst reduced at 200 °C (Figure 5, profile 2) indicating that the formation of hydride is inhibited for PdIn catalysts even after mild reduction. The TPHD profiles for the catalysts reduced at higher temperature do not show any signs of H_2_ evolution.

### 3.2. Catalytic Hydrogenation of DPA

#### 3.2.1. Effect of Reduction Temperature on the Activity of PdIn Catalysts

**Figure 6:**
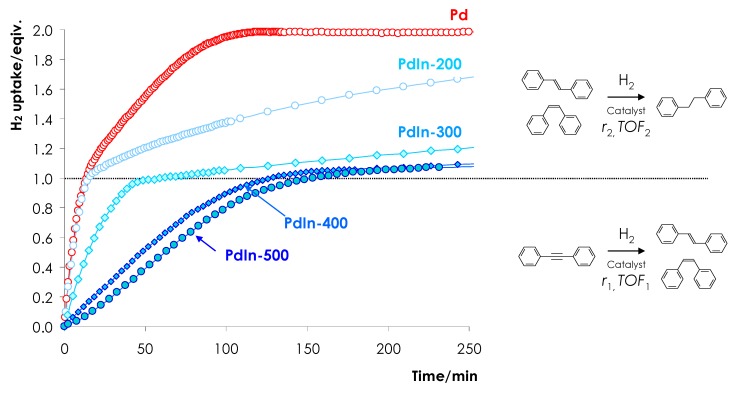
Effect of reduction temperature on the kinetics of DPA hydrogenation for Pd/α-Al_2_O_3_ and PdIn/α-Al_2_O_3_ catalysts reduced at 200–500 °C.

Figure 6 depicts characteristic kinetic profile of H_2_ uptake as a function of the reaction time in the course of liquid-phase DPA hydrogenation over the reference Pd/α-Al_2_O_3_ and PdIn/α-Al_2_O_3_ reduced at different temperatures. The data can be interpreted in accordance with the classical two-stage mechanism of substituted alkynes hydrogenation [55,56]. At the first stage, the predominant diphenylacetylene-to-stilbene hydrogenation occurs, which results in the uptake of 1 equiv of H_2_. At the second stage the resulting stilbene is hydrogenated to diphenylethane with the uptake of the second H_2_ equivalent.

Kinetic profiles show typical downward bending after consumption of 1 H_2_ equivalent. The bending is attributed to a decrease in the hydrogenation rate after completion of alkyne hydrogenation. The results are in a good agreement with previously reported data on the DPA hydrogenation over Pd catalysts [57,58].

Comparing the kinetic profiles (Figure 6) and the hydrogenation rates (Table 1) we can conclude that the rates of the triple bond hydrogenation (<1 equiv. H_2_ uptake, *r*_1_) for Pd/Al_2_O_3_ and PdIn/Al_2_O_3_ reduced at 200 °C (PdIn-200) are essentially identical. Thus *r*_1_ = 4.39 mmol/(g_cat_ min) for monometallic Pd and 4.02 mmol/(g_cat_ min) for PdIn-200 (Table 1, Figure 6).

It is noteworthy that the H_2_ uptake profile for PdIn-200 exhibits more pronounced downward bending as compared to the profile of the monometallic Pd catalyst (Figure 6). This observation indicates that the rate of the second stage (>1 equiv. H_2_ uptake, *r*_2_) notably decreases over PdIn-200 as compared to monometallic Pd catalyst and *r*_1_/*r*_2_ ratio increases from 7.1 to 20.7. The observed decrease in the hydrogenation rate is attributable to the suppression of PdH*_x_* hydride phase (see Figure 5) due to the intensive formation of PdIn alloy enriched in Pd as indicated by XRD data (see Insert of Figure 2, pattern 4). Note, that similar effect was discussed by several authors for Pd catalysts modified with Zn [8], Ag [30,31], Sn, Sb, Ge, Pb [53], Ga [59]. In our previous study, this effect was also observed for PdIn/SiO_2_ catalyst [51].

For the catalysts reduced at 300 and 400 °C, kinetic profiles of H_2_ uptake point to a decrease in DPA hydrogenation rate and *r*_1_ decreases to 1.40 and 0.46 mmol/(g_cat_ min), respectively. Since the sizes of metal particles are similar in monometallic Pd and PdIn-400 according to TEM data (compare Figure 3 and Figure 4d), the 10-fold lower activity of PdIn-400 cannot be assigned only to a difference in metal dispersion. Presumably activity decrease is attributable to the formation of Pd_1_In_1_ intermetallic nanoparticles, which predominate in PdIn-400. It is conceivable, since a part of PdIn surface is occupied by inactive In atoms. Moreover, formation of Pd_1_In_1_ intermetallic nanoparticles could decrease the activity of Pd sites by lowering its *d*-band energy thus reducing the adsorption energy of the alkyne and alkene [60].

It is remarkable that the formation of Pd_1_In_1_ intermetallic nanoparticles results in the preferential decrease in reaction rate on the second reaction stage, and the *r*_1_/*r*_2_ ratio increases from 35.0 for PdIn-300 to ~45–48 for PdIn-400–PdIn-600. These data are in a correlation with XRD and TEM results and can be explained by the complete transformation of PdIn solid solution into the IMCs at 400 °C.

The specific hydrogenation kinetics on the PdIn catalysts reduced at 400–600 °C are of considerable practical interest, since the predominant decrease in the reaction rate on the second stage of alkene hydrogenation allows one to control effectively the course of the reaction in a batch reactor and to stop the hydrogenation after the alkyne-to-olefin conversion preventing the loss of the desirable product.

#### 3.2.2. Selectivity to Olefin Formation

**Figure 7:**
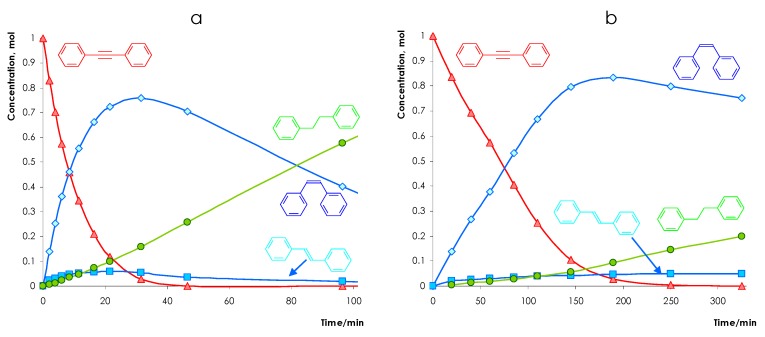
Reaction profiles for the competitive DPA hydrogenation over Pd/α-Al_2_O_3_ (**a**) and PdIn/α-Al_2_O_3_ catalysts reduced at 500 °C (**b**).

The typical dependencies of the reaction mixture composition on the reaction time are shown in Figure 7 for the Pd/α-Al_2_O_3_ and Pd-In/α-Al_2_O_3_ reduced at 500 °C (PdIn-500).

The general volcano-type shapes of the ‘stilbene concentration—reaction time’ curves imply the sequential mechanism of the DPA hydrogenation to stilbene followed by the hydrogenation of stilbene to diphenylethane. However, the dependencies for the Pd and PdIn catalysts differs each other significantly. For the monometallic catalyst, diphenylethane appears in the reaction products even at low DPA conversions and at the moment of complete DPA conversion diphenylethane concentration exceeds 25–27% (Figure 7a). For PdIn-500 catalyst the concentration of diphenylethane formed on the first stage in the presence of an initial alkyne is significantly lower and does not exceed 14% after the total DPA conversion (Figure 7b). Comparison of the concentration patterns of both bimetallic and monometallic samples reveals that the maximum yield of the desired stilbene increases from ca. 73–75% for monometallic Pd to 84–85% for the bimetallic PdIn-500 sample. The data obtained show that over bimetallic catalysts a contribution of the total hydrogenation to the overall process decreases. This leads to an increase in the yield of the desired alkene intermediate and, as a consequence, improves selectivity of the process.

**Figure 8:**
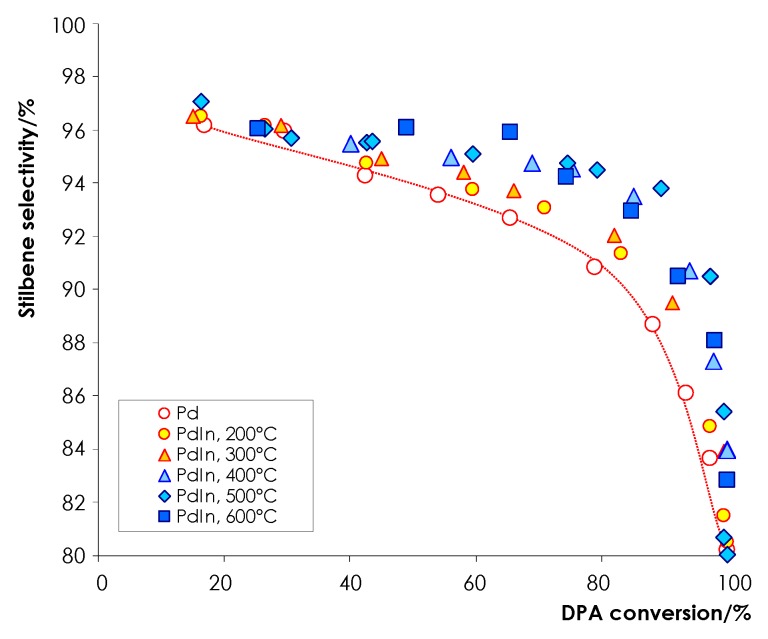
Stilbene selectivity as a function of DPA conversion over mono- and bimetallic catalysts in the selective DPA hydrogenation. *P*_H2_ = 10 bar, *T* = 25 °C, [*C_DPA_*] = 0.160 mol L^−1^, *m*_cat_ = 5.0 mg; n-hexane as a solvent.

It is informative to analyze the dependence of the selectivity to stilbene as a function of the DPA conversion (Figure 8). The analysis demonstrates that at low DPA conversions (<40%) selectivity values are identical for both Pd and PdIn samples. This observation suggests that a contribution of a direct hydrogenation of the alkene intermediate to the total process is insignificant and the forming stilbene is predominantly desorbs to the solution. With the gradual increase in DPA conversion, the selectivity decreases continuously, because at high concentrations stilbene replaces the parent alkyne from the catalyst surface, which leads to the formation of undesirable alkane. However, at high DPA conversion, the alkene selectivity of PdIn/Al_2_O_3_ is significantly higher as compared to Pd sample. For example, at the DPA conversion >90%, the selectivity to stilbene over PdIn/Al_2_O_3_ is ca. 88–92%, whereas the selectivity for the monometallic sample is below 84–86%. It should be mentioned that the analysis of the data in Figure 8 indicates that the selectivity tends to improve as the reduction temperature increases. Comparison of the selectivity data with the characterization results allows us to suggest that the selectivity improvement results from several factors. Better selectivity of the catalyst reduced at 200 °C stems, presumably, from the suppression of PdH*_x_* formation due to formation of PdIn alloy, as indicated by XRD and TPHD data. Further selectivity improvement observed for the catalyst reduced at higher temperatures is attributable to the progressive formation of Pd_1_In_1_ nanoparticles predominating in the catalysts reduced at 400–600 °C. The suggestion is in good agreement with the data on the excellent selectivity of PdGa [6,10] and PdIn IMCs in selective acetylene hydrogenation [15], and the observed selectivity improvement can be explained by isolation of Pd sites by inactive In atoms [13,15], and modification of their electronic structure similarly to PdGa IMC [10,61].

## 4. Conclusions

**Figure 9:**
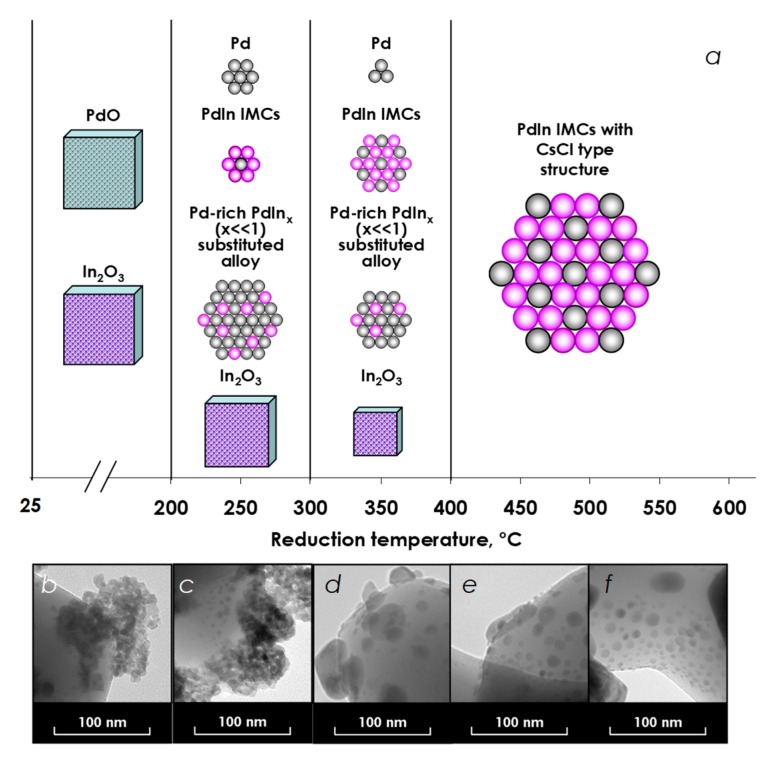
Transformation of the PdIn/α-Al_2_O_3_ catalyst structure upon stepwise increase of reduction temperature (**a**). Corresponding TEM micrographs, collected for unreduced PdIn/α-Al_2_O_3_ (**b**) andPdIn/α-Al_2_O_3_ reduced at 200 °C (**c**); 300 °C (**d**); 400 °C (**e**); and 500 °C (**f**).

Detailed study of PdIn nanoparticles formation by combination of TEM, XRD, and the temperature-programmed Pd hydride decomposition revealed several distinct stages of transformations in the catalyst structure and performance (Figure 9). Mild reduction by hydrogen at 200 °C results in the intensive formation of PdIn*_x_* substitutional alloy rich in Pd and the pronounced suppression of Pd hydride formation. The rate of DPA triple bond hydrogenation over PdIn-200 catalyst is identical to that over the reference monometallic Pd catalyst, while the rate of the subsequent double bond hydrogenation is significantly reduced facilitating effective kinetic control of semi-hydrogenation.

The increase in the reduction temperature to 300 °C leads to the appearance of Pd_1_In_1_ intermetallic nanoparticles coexisting with PdIn*_x_* substitutional alloy clusters. The rate of the triple bond hydrogenation on PdIn-300 decreases by ~3 times. However, the rate of the subsequent double bond hydrogenation is reduced by a factor of ~15, thus improving the efficiency of the kinetic control.

Neither monometallic Pd or In nor other intermetallic structures were found after reduction at 400–600 °C, indicating that the catalyst contains Pd_1_In_1_ nanoparticles of identical composition. 

The formation of Pd_1_In_1_ intermetallic nanoparticles is accomplished upon reduction at 400 °C, and neither monometallic Pd or In nor other intermetallic structures are detected in the catalysts reduced at 400–600 °C. These data allow us to conclude that the proposed preparation method enables to obtain the catalysts containing supported intermetallic nanoparticles with uniform Pd_1_In_1_ composition. The catalysts exhibit excellent selectivity in the liquid-phase DPA (yield > 85%), though the hydrogenation rate is lowered by a factor of 10 as compared to Pd-200 catalyst. The selectivity data are in a good agreement with the data on the high selectivity of PdGa [6,10] and PdIn IMCs in the gas-phase selective acetylene hydrogenation [15] and can be explained by: (1) suppression of Pd hydride formation, (2) isolation of Pd sites by inactive In atoms, and (3) modification of their electronic structure.

## Figures and Tables

**Table 1:** Kinetic parameters of synthesized catalysts in liquid-phase DPA hydrogenation.

Catalyst	*r*_1_	*r*_2_	*r*_1_/*r*_2_
	mmol/(g_cat_ min)		
Pd	4.39	0.621	7.1
PdIn-200	4.02	0.194	20.7
PdIn-300	1.40	0.040	35.0
PdIn-400	0.46	0.0103	44.7
PdIn-500	0.42	0.0088	47.7
PdIn-600	0.45	0.0095	47.4

*P*_H2_ = 10 bar, T = 25 °C, m_cat_ = 5 mg, *n*-hexane as solvent.

## References

[1] Armbrüster M., Horváth I.T. (2011). Intermetallic Compounds in Catalysis. Encyclopedia of Catalysis.

[2] Furukawa S., Komatsu T. (2017). Intermetallic Compounds: Promising Inorganic Materials for Well-Structured and Electronically Modified Reaction Environments for Efficient Catalysis. ACS Catal..

[3] Zafeiratos S., Piccinin S., Teschner D. (2012). Alloys in catalysis: Phase separation and surface segregation phenomena in response to the reactive environment. Catal. Sci. Technol..

[4] Komatsu T., Furukawa S. (2015). Intermetallic Compound Nanoparticles Dispersed on the Surface of Oxide Support as Active and Selective Catalysts. Mater. Trans..

[5] Penner S., Armbrüster M. (2015). Formation of Intermetallic Compounds by Reactive Metal–Support Interaction: A Frequently Encountered Phenomenon in Catalysis. ChemCatChem.

[6] Armbrüster M., Schlögl R., Grin Y. (2014). Intermetallic compounds in heterogeneous catalysis—A quickly developing field. Sci. Technol. Adv. Mater..

[7] Lorenz H., Rameshan C., Bielz T., Memmel N., Stadlmayr W., Mayr L., Zhao Q., Soisuwan S., Klötzer B., Penner S. (2013). From Oxide-Supported Palladium to Intermetallic Palladium Phases: Consequences for Methanol Steam Reforming. ChemCatChem.

[8] Ananikov V.P., Eremin D.B., Yakukhnov S.A., Dilman A.D., Levin V.V., Egorov M.P., Karlov S.S., Kustov L.M., Tarasov A.L., Greish A.A. (2017). Organic and hybrid systems: From science to practice. Mendeleev Commun..

[9] Föttinger K. (2013). PdZn based catalysts: Connecting electronic and geometric structure with catalytic performance. Catalysis.

[10] Kovnir K., Armbrüster M., Teschner D., Venkov T.V., Jentoft F.C., Knop-Gericke A., Grin Y., Schlögl R. (2007). A new approach to well-defined, stable and site-isolated catalysts. Sci. Technol. Adv. Mater..

[11] Arnold H., Döbert F., Gaube J., Ertl G., Knözinger H., Schüth F., Weitkamp J. (2008). Selective Hydrogenation of Hydrocarbons. Handbook of Heterogeneous Catalysis.

[12] Molnár Á., Sárkány A., Varga M. (2001). Hydrogenation of carbon–carbon multiple bonds: Chemo-, regio- and stereo-selectivity. J. Mol. Catal. A Chem..

[13] Stakheev A.Y., Smirnova N.S., Krivoruchenko D.S., Baeva G.N., Mashkovsky I.S., Yakushev I.A., Vargaftik M.N. (2017). Single-atom Pd sites on the surface of Pd-In nanoparticles supported on γ-Al_2_O_3_: A CO-DRIFTS study. Mendeleev Commun..

[14] Markov P.V., Bragina G.O., Baeva G.N., Mashkovskii I.S., Rassolov A.V., Yakushev I.A., Vargaftik M.N., Stakheev A.Y. (2016). Supported Catalysts Based on Pd–In Nanoparticles for the Liquid-Phase Hydrogenation of Terminal and Internal Alkynes: 2. Catalytic Properties. Kinet. Catal..

[15] Feng Q., Zhao S., Wang Y., Dong J., Chen W., He D., Wang D., Yang J., Zhu Y., Zhu H. (2017). Isolated Single-Atom Pd Sites in Intermetallic Nanostructures: High Catalytic Selectivity for Semihydrogenation of Alkynes. J. Am. Chem. Soc..

[16] Burueva D.B., Kovtunov K.V., Bukhtiyarov A.V., Barskiy D.A., Prosvirin I.P., Mashkovsky I.S., Baeva G.N., Bukhtiyarov V.I., Stakheev A.Y., Koptyug I.V. (2018). Selective Single-Site Pd-In Hydrogenation Catalyst for Production of Enhanced Magnetic Resonance Signals using Parahydrogen. Chem. Eur. J..

[17] Lorenz H., Turner S., Lebedev O.I., van Tendeloo G., Klötzer B., Rameshan C., Pfaller K., Penner S. (2010). Pd–In_2_O_3_ interaction due to reduction in hydrogen: Consequences for methanol steam reforming. Appl. Catal. A.

[18] Smirnova N.S., Shlyapin D.A., Mironenko O.O., Anoshkina E.A., Temerev V.L., Shitova N.B., Kochubey D.I., Tsyrul’nikov P.G. (2012). EXAFS study of Pd/Ga_2_O_3_ model catalysts of selective liquid-phase hydrogenation of acetylene to ethylene. J. Mol. Catal. A Chem..

[19] Vilé G., Dähler P., Vecchietti J., Baltanás M., Collins S., Calatayud M., Bonivardi A., Pérez-Ramírez J. (2015). Promoted ceria catalysts for alkyne semi-hydrogenation. J. Catal..

[20] Neumann M., Teschner D., Knop-Gericke A., Reschetilowski W., Armbrüster M. (2016). Controlled synthesis and catalytic properties of supported In–Pd intermetallic compounds. J. Catal..

[21] Zhou H., Yang X., Li L., Liu X., Huang Y., Pan X., Wang A., Li J., Zhang T. (2016). PdZn Intermetallic Nanostructure with Pd-Zn-Pd ensembles for Highly Active and Chemoselective Semi-hydrogenation of Acetylene. ACS Catal..

[22] García-Trenco A., Regoutz A., White E.R., Payne D.J., Shaffer M.S.P., Williams C.K. (2018). PdIn intermetallic nanoparticles for the Hydrogenation of CO_2_ to Methanol. Appl. Catal. B.

[23] Wu Z., Wegener E.C., Tseng H.-T., Gallagher J.R., Harris J.W., Diaz R.E., Ren Y., Ribeiro F.H., Miller J.T. (2016). Pd–In intermetallic alloy nanoparticles: Highly selective ethane dehydrogenation catalysts. Catal. Sci. Technol..

[24] Armbrüster M., Behrens M., Föttinger K., Friedrich M., Gaudry É., Matam S.K., Sharma H.R. (2013). The Intermetallic Compound ZnPd and Its Role in Methanol Steam Reforming. Catal. Rev. Sci. Eng..

[25] Predel B., Madelung O. (1997). Phase Equilibria, Crystallographic and Thermodynamic Data of Binary Alloys. Landolt-Börnstein, New Series IV/5G (1997), New Series IV/5G.

[26] Okamoto H. (2003). In–Pd (Indium–Palladium). J. Phase Equilib..

[27] Penner S., Wang D., Su D.S., Rupprechter G., Podloucky R., Schlögl R., Hayek K. (2003). Platinum nanocrystals supported by silica, alumina and ceria: Metal–support interaction due to high-temperature reduction in hydrogen. Surf. Sci..

[28] Stolarov I.P., Yakushev I.A., Churakov A.V., Cherkashina N.V., Smirnova N.S., Khramov E.V., Zubavichus Y.V., Khrustalev V.N., Markov A.A., Klyagina A.P. (2018). Heterometallic Palladium(II)−Indium(III) and −Gallium(III) Acetate-Bridged Complexes: Synthesis, Structure, and Catalytic Performance in Homogeneous Alkyne and Alkene Hydrogenation. Inorg. Chem..

[29] Markov P.V., Bragina G.O., Rassolov A.V., Baeva G.N., Mashkovsky I.S., Murzin V.Y., Zubavichus Y.V., Stakheev A.Y. (2016). Pd–Cu catalyst prepared from heterobimetallic PdCu_2_(OAc)_6_: An XRD-EXAFS study and activity/selectivity in the liquid-phase hydrogenation of a C≡C bond. Mendeleev Commun..

[30] Rassolov A.V., Markov P.V., Bragina G.O., Baeva G.N., Mashkovskii I.S., Yakushev I.A., Vargaftik M.N., Stakheev A.Y. (2016). Catalytic Properties of Nanostructured Pd–Ag Catalysts in the Liquid-Phase Hydrogenation of Terminal and Internal Alkynes. Kinet. Catal..

[31] Rassolov A.V., Markov P.V., Bragina G.O., Baeva G.N., Krivoruchenko D.S., Mashkovskii I.S., Yakushev I.A., Vargaftik M.N., Stakheev A.Y. (2016). Formation of Pd–Ag Nanoparticles in Supported Catalysts Based on the Heterobimetallic Complex PdAg_2_(OAc)_4_(HOAc)_4_. Kinet. Catal..

[32] Rassolov A.V., Krivoruchenko D.S., Medvedev M.G., Mashkovsky I.S., Stakheev A.Y., Svitanko I.V. (2017). Diphenylacetylene hydrogenation on a PdAg/Al_2_O_3_ single-atom catalyst: An experimental and DFT study. Mendeleev Commun..

[33] Ichimura K. (2000). Photoalignment of Liquid-Crystal Systems. Chem. Rev..

[34] Furukawa S., Yokoyama A., Komatsu T. (2014). Efficient Catalytic System for Synthesis of trans-Stilbene from Diphenylacetylene Using Rh-Based Intermetallic Compounds. ACS Catal..

[35] Halim M., Samuel I.D.W., Pillow J.N.G., Monkman A.P., Burn P.L. (1999). Control of Colour and Charge Injection in Conjugated Dendrimer/Polypyridine Bilayer LEDs. Synth. Met..

[36] Blaser H.-U., Schnyder A., Steiner H., Rossler F., Baumeister P., Ertl G., Knözinger H., Schüth F., Weitkamp J. (2008). Selective Hydrogenation of Functionalized Hydrocarbons. Handbook of Heterogeneous Catalysis.

[37] Gavrikov A.V., Koroteev P.S., Dobrokhotova Z.V., Ilyukhin A.B., Efimov N.N., Kirdyankin D.I., Bykov M.A., Ryumin M.A., Novotortsev V.M. (2015). Novel heterometallic polymeric lanthanide acetylacetonates with bridging cymantrenecarboxylate groups—Synthesis, magnetism and thermolysis. Polyhedron.

[38] Markov P.V., Bragina G.O., Baeva G.N., Tkachenko O.P., Mashkovskii I.S., Yakushev I.A., Kozitsyna N.Y., Vargaftik M.N., Stakheev A.Y. (2015). Pd–Cu Catalysts from Acetate Complexes in Liquid-Phase Diphenylacetylene Hydrogenation. Kinet. Catal..

[39] Mashkovsky I.S., Markov P.V., Bragina G.O., Rassolov A.V., Baeva G.N., Stakheev A.Y. (2017). Intermetallic Pd_1_–Zn_1_ Nanoparticles in the Selective Liquid-Phase Hydrogenation of Substituted Alkynes. Kinet. Catal..

[40] Chauruka S.R., Hassanpour A., Brydson R., Roberts K.J., Ghadiri M., Stitt H. (2015). Effect of mill type on the size reduction and phase transformation of gamma alumina. Chem. Eng. Sci..

[41] Matori K.A., Wah L.C., Hashim M., Ismail I., Mohd Zaid M.H. (2012). Phase Transformations of α-Alumina Made from Waste Aluminum via a Precipitation Technique. Int. J. Mol. Sci..

[42] Li Z., Wu K., Cao J., Wang Y. (2017). Controlled synthesis of α-Al_2_O_3_ via the hydrothermalpyrolysis method. IOP Conf. Ser. Mater. Sci. Eng..

[43] Akhtar M.J., Ahamed M., Kumar S., Majeed Khan M.A., Ahmad J., Alrokayan S.A. (2012). Zinc oxide nanoparticles selectively induce apoptosis in human cancer cells through reactive oxygen species. Int. J. Nanomed..

[44] Mashkovsky I.S., Markov P.V., Bragina G.O., Baeva G.N., Bukhtiyarov A.V., Prosvirin I.P., Bukhtiyarov V.I., Stakheev A.Y. (2017). Formation of Supported Intermetallic Nanoparticles in the Pd–Zn/α-Al_2_O_3_ Catalyst. Kinet. Catal..

[45] Mashkovsky I.S., Markov P.V., Bragina G.O., Baeva G.N., Rassolov A.V., Bukhtiyarov A.V., Prosvirin I.P., Bukhtiyarov V.I., Stakheev A.Y. (2018). PdZn/α-Al_2_O_3_ catalyst for liquid-phase alkyne hydrogenation: Details of «solid-state alloy—Intermetallics» transformation. Mendeleev Commun..

[46] Baylet A., Marécot P., Duprez D., Castellazzi P., Groppi G., Forzatti P. (2011). In situ Raman and in situ XRD analysis of PdO reduction and Pd^0^ oxidation supported on γ-Al_2_O_3_ catalyst under different atmospheres. Phys. Chem. Chem. Phys..

[47] Chitturi K.L., Yaramma A., Merugu R., Dachepalli R., Kandhadi J. (2016). Synthesis and Characterisation of In_2_O_3_ Nanoparticles from *Astragalus gummifer*. Adv. Nanopart..

[48] Choi Y.I., Kim S.K., Lee S.W., Sohn Y. (2016). Metallic indium spheres by the anaerobic ethanol oxidation of indium oxide. J. Alloys Compd..

[49] Bukhtiyarov V.I., Zaikovskii V.I., Kashin A.S., Ananikov V.P. (2016). Modern electron microscopy in the study of chemical systems at the boundary of organic synthesis and catalysis. Russ. Chem. Rev..

[50] Armbrüster M., Behrens M., Cinquini F., Föttinger K., Grin Y., Haghofer A., Klötzer B., Knop-Gericke A., Lorenz H., Ota A. (2012). How to Control the Selectivity of Palladium-based Catalysts in Hydrogenation Reactions: The Role of Subsurface Chemistry. ChemCatChem.

[51] Markov P.V., Bragina G.O., Baeva G.N., Tkachenko O.P., Mashkovsky I.S., Yakushev I.A., Vargaftik M.N., Stakheev A.Y. (2016). Supported Catalysts Based on Pd–In Nanoparticles for the Liquid-Phase Hydrogenation of Terminal and Internal Alkynes: 1. Formation and Structure. Kinet. Catal..

[52] Neri G., Musolino M.G., Milone C., Pietropaolo D., Galvagno S. (2001). Particle size effect in the catalytic hydrogenation of 2,4-dinitrotoluene over Pd/C catalysts. Appl. Catal. A Gen..

[53] Aduriz H.R., Bodnariuk P., Coq B., Figueras F. (1989). Alumina-Supported Bimetallics of Palladium Alloyed with Germanium, Tin, Lead, or Antimony from Organometallic Precursors. I. Preparation and characterization. J. Catal..

[54] Cao Y., Sui Z.J., Zhu Y., Zhou X., Chen D. (2017). Selective Hydrogenation of Acetylene over Pd-In/Al_2_O_3_ Catalyst: Promotional Effect of Indium and Composition-dependent Performance. ASC Catal..

[55] Bond G.C. (2005). Metal-Catalysed Reactions of Hydrocarbons.

[56] Markov P.V., Bragina G.O., Rassolov A.V., Mashkovsky I.S., Baeva G.N., Tkachenko O.P., Yakushev I.A., Vargaftik M.N., Stakheev A.Y. (2016). Performance of the bimetallic Pd-In catalyst in the selective liquid-phase hydrogenation of internal and terminal alkynes. Mendeleev Commun..

[57] Choudary B.M., Lakshmi Kantam M., Mahender Reddy N., Koteswara Rao K., Haritha Y., Bhaskar V., Figueras F., Tuel A. (1999). Hydrogenation of acetylenics by Pd-exchanged mesoporous materials. Appl. Catal. A Gen..

[58] Marín-Astorga N., Alvez-Manoli G., Reyes P. (2005). Stereoselective hydrogenation of phenyl alkyl acetylenes on pillared clays supported palladium catalysts. J. Mol. Catal. A Chem..

[59] Ota A., Armbrüster M., Behrens M., Rosenthal D., Friedrich M., Kasatkin I., Girgsdies F., Zhang W., Wagner R., Schlögl R. (2011). Intermetallic Compound Pd_2_Ga as a Selective Catalyst for the Semi-Hydrogenation of Acetylene: From Model to High Performance Systems. J. Phys. Chem. C.

[60] Liu J., Zhu Y., Liu C., Wang X., Cao C., Song W. (2017). Excellent Selectivity with High Conversion in the Semihydrogenation of Alkynes using Palladium-Based Bimetallic Catalysts. ChemCatChem.

[61] Wencka M., Hahne M., Kocjan A., Vrtnik S., Koželj P., Korže D., Jagličić Z., Sornić M., Popčević P., Ivkov J. (2014). Physical properties of the InPd intermetallic catalyst. Intermetallics.

